# Kola

**Published:** 1886-07-20

**Authors:** 


**Kola.**—The kola of commerce is the seed of the fruit of the *sterculia acuminata*, a tree from thirty to sixty feet in height, indigenous to the west coast of Africa. The physiological effects attributed to the drug are somewhat similar to those ascribed to coca.

